# The role of polarisation of circulating tumour cells in cancer metastasis

**DOI:** 10.1007/s00018-019-03169-3

**Published:** 2019-06-19

**Authors:** Mathias Heikenwalder, Anna Lorentzen

**Affiliations:** 1 Divison of Chronic Inflammation and Cancer, German Cancer Research Center (DKFZ), 69120 Heidelberg, Germany; 2 Department of Molecular Biology and Genetics, Aarhus University, 8000 Aarhus, Denmark

**Keywords:** Cancer, Single-cell polarity, CTC, Adhesion, Attachment, Actin

## Abstract

Metastasis is the spread of cancer cells from a primary tumour to a distant site of the body. Metastasising tumour cells have to survive and readjust to different environments, such as heterogeneous solid tissues and liquid phase in lymph- or blood circulation, which they achieve through a high degree of plasticity that renders them adaptable to varying conditions. One defining characteristic of the metastatic process is the transition of tumour cells between different polarised phenotypes, ranging from differentiated epithelial polarity to migratory front–rear polarity. Here, we review the polarisation types adopted by tumour cells during the metastatic process and describe the recently discovered single-cell polarity in liquid phase observed in circulating tumour cells. We propose that single-cell polarity constitutes a mode of polarisation of the cell cortex that is uncoupled from the intracellular polarisation machinery, which distinguishes single-cell polarity from other types of polarity identified so far. We discuss how single-cell polarity can contribute to tumour metastasis and the therapeutic potential of this new discovery.

## Introduction

Metastasis is the leading cause of mortality in cancer patients [1, 2]. However, no specific anti-metastatic treatments are available for clinical use due to the difficulty of identifying essential steps during the metastatic process that can be attacked pharmacologically to reduce or prevent metastasis in cancer patients. Metastasis is a complex multistep process and many of the molecular details are not fully elucidated. The metastatic process is often described by the “invasion-metastasis cascade” [3, 4], which comprises dedifferentiation, dissociation and local invasion of primary tumour cells; intravasation into blood or lymph vessels; survival and transport of circulating tumour cells (CTCs) in the blood; attachment or arrest in micro-vessels of distant organs; adhesion and extravasation; and survival and growth of metastases. At each of these steps, metastasising cells need to be able to adapt to the changing environmental conditions. Tumour cells thus require high levels of plasticity in terms of extracellular communication with the microenvironment, transcriptional programmes, intracellular signalling, cell morphology or modes of migration in order to successfully establish metastases [5–8].

Metastasis is also characterised by polarisation plasticity, which is the ability of tumour cells to undergo various depolarisation or repolarisation events and to adopt different polarity types by rewiring of their polarity machineries [9, 10]. During dedifferentiation, primary tumour cells lose their epithelial polarity and cell–cell adhesions and acquire invasive properties in the process of epithelial-to-mesenchymal transition (EMT) [11–14]. This allows tumour cells to adopt a migratory phenotype characterised by front–rear polarisation to locally invade the tumour-surrounding tissue [15, 16] and to intravasate into lymph or blood vessels [17–19]. In circulation, tumour cells maintain a basic type of cortical polarity [20] that can facilitate attachment to the endothelial wall and repolarisation necessary for adhesion and extravasation. The cortical polarity, that is either maintained or newly established during adhesion [21], transitions into front–rear polarity of migratory cells required for transmigration through the vessel wall and interstitial migration. Eventually, metastasising cells need to re-acquire epithelial polarity to establish and grow tumours at secondary sites, in a process termed mesenchymal-to-epithelial transition (MET) [22–25].

In the following sections, we present a brief overview over the different types of polarity that tumour cells can adopt during the metastatic process, how cells connect molecular polarity modules to achieve these types of polarity and the underlying regulatory processes. We specifically focus on the recently discovered polarisation of tumour cells during circulation termed single-cell polarity in liquid phase [20]. Single-cell polarity was recently discovered in tumour cells in suspension in vitro and in CTCs from cancer patients. It constitutes a cortical type of polarisation characterised by accumulation of the actin cytoskeleton, the plasma membrane and linker proteins at one pole of single cells in liquid phase. Single-cell polarity has been demonstrated to enhance unspecific attachment and advance adhesion of tumour cells, thereby increasing their potential for metastatic seeding. We explore single-cell polarity in relation to other types of polarity relevant for cancer metastasis and the potential processes underlying transitions between polarity types. Finally, we discuss possible mechanisms by which such polarisation events can contribute to tumour metastasis and could be targeted therapeutically.

## Different types of cellular polarity

### Epithelial polarity

**Fig. 1:**
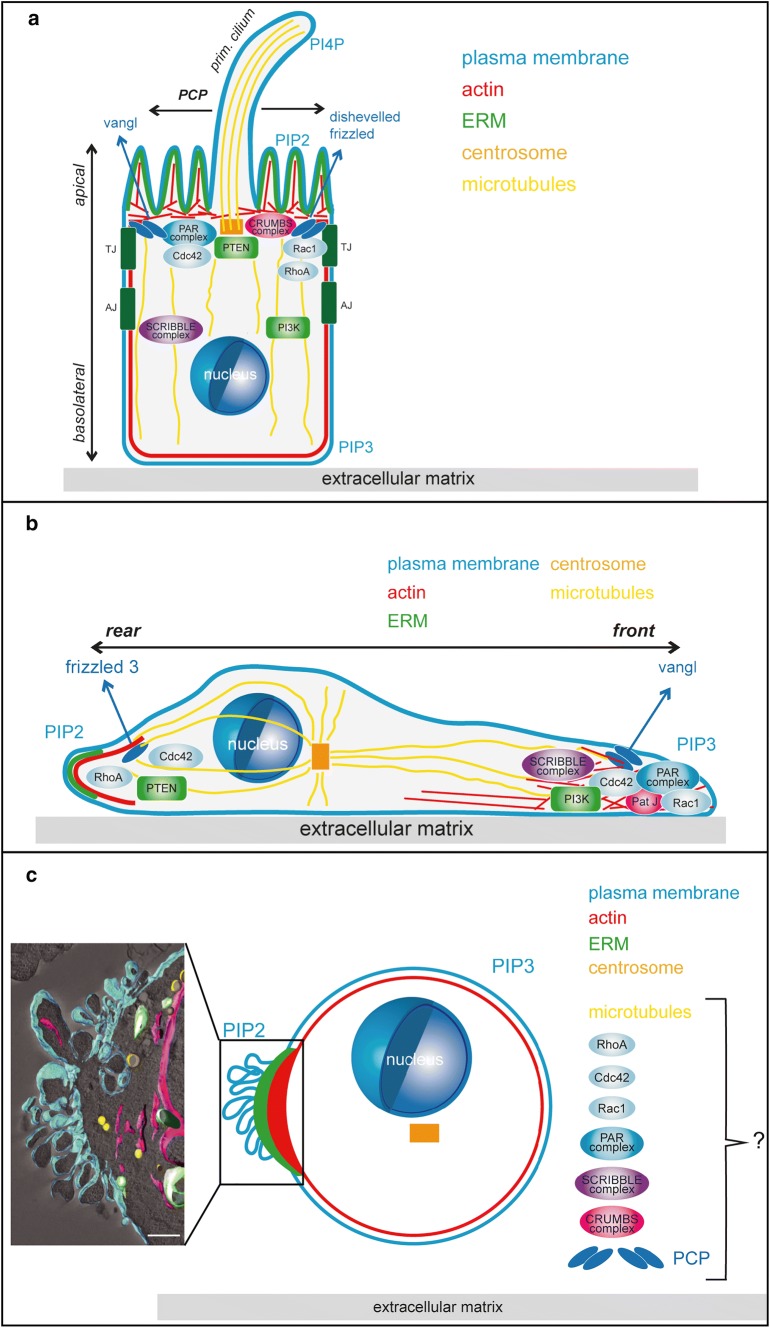
Polarisation states of tumour cells. The illustrations depict the localisation of some of the main polarity regulating modules in an epithelial cell displaying apical–basal polarity and planar cell polarity (PCP) (**a**), a migrating cell displaying front–rear polarity (**b**) or a cell in liquid phase displaying single-cell polarity (**c**). The inset in **c** shows a tomogram of the pole of an SkMel2 cell in suspension, adapted from [20], showing plasma membrane (cyan), ER and nuclear envelope (magenta), mitochondria (green) and lipid droplets (yellow). Scale bar 1 μm. While the molecules sustaining the different polarised phenotypes are the same in the different types of polarity, their localisation and interconnections differ. For details see main text. *TJ* tight junction, *AJ* adherens junction, *PI3K* phosphoinositide-3-kinase, *PIP2* phosphatidylinositol 4,5 bisphosphate, *PIP3* phosphatidylinositol 3,4,5 trisphosphate

Cells organised in epithelia establish two distinct surfaces, an apical side oriented towards the lumen and a basal side that contacts the extracellular matrix of the basal lamina (Fig. 1a). The lateral sides of epithelial cells are forming close contacts via tight junctions and adherens junctions. Apical–basal polarity is characterised by asymmetrical distribution of cellular components such as proteins, phospholipids, mRNAs, cytoskeletons, the membrane trafficking, secretory and recycling machinery as well as organelles [26].

This specific architecture outlined above is tightly regulated by three interconnected polarity complexes (Fig. 1a): the partitioning defective (PAR) complex, the CRUMBS complex and the SCRIBBLE complex [26, 27]. The PAR complex, which contains the proteins Par3, Par6, atypical protein kinase C (aPKC) and cell division cycle 42 (Cdc42, a member of the Rho-family of small GTPases) is localised in the apical area, where it is involved in establishing the apical domain [28–30]. The protein Par3 is involved in tight junction assembly by controlling Rac1 activity [31]. The CRUMBS complex includes the protein Crumbs, protein associated with Lin7 1 (Pals 1) and Pals1-associated tight junction protein (PATJ) and is localised at the apical membrane where it is responsible for assembly and stabilisation of tight junctions [32, 33] and the stability of epithelial polarity [34]. The SCRIBBLE complex contains the proteins Scribble, lethal giant larvae (Lgl) and disc large homolog (Dlg) and is localised at the basolateral side where it is involved in establishing the basolateral domain and in suppressing extension of the apical domain [35, 36].

In addition to apical–basal polarisation, epithelia are also polarised in the horizontal direction orthogonal to the apical–basal axis, by planar cell polarity (PCP) [37–40] to coordinate cell functions or cilia positioning along the epithelial plane (Fig. 1a). Core components of the PCP include the non-canonical Wnt receptor Frizzled, Dishevelled, Prickle, Vangl1 and Vangl2. Complexes of PCP components are asymmetrically localised along the apical cell surface to form a proximal and a distal side along the lateral, epithelial surface.

Polarity complexes do not act as separate entities but as highly integrated networks that mutually regulate each other’s function, localisation and activity (reviewed in [9, 10]) in cooperation with other key polarity regulators and components such as lipid kinases/phosphatases [10, 41], Rab- [42] and Rho-family GTPases [43–45] and the actin-, microtubule- and intermediate filament cytoskeletons [26, 46, 47].

In epithelia, the apical plasma membrane is enriched in phosphatidylinositol 4,5 bisphosphate (PIP2), the basolateral plasma membrane with phosphatidylinositol 3,4,5 trisphosphate (PIP3) (Fig. 1a). This asymmetric distribution of membrane lipids is established by local activities of phosphoinositide-3-kinases (PI3-kinases) [48–50], phosphatase and tensin homolog (PTEN) [51, 52] and additional lipid kinases and phosphatases [53]. The polarised distribution of PIP2 and PIP3 in apical and basolateral membranes furthermore underlies the regulation by polarity complexes and Rho GTPase signalling networks that restrict the subcellular localisation of PI3K to the lateral side and PTEN to the apical side [54, 55]. Conversely, PIP2 and PIP3 provide binding platforms for a range of proteins required to establish or maintain epithelial polarity [56] such as Annexin 2, which binds to PIP2 at the apical surface, where it binds and recruits Cdc42, which can then recruit the other Par complex proteins [54].

Small GTPases of the Rab-family regulate membrane and cargo transport pathways. Especially the Rab11 subfamily has been implicated in the regulation of epithelial polarity [42]. Rab11a together with Rab8a targets Par3 and Cdc42 activity to an apical membrane initiation site for the generation of an apical surface during lumen formation [57]. In addition, Rab11 is involved in trafficking and recycling to maintain the apical [58] and basolateral [59] domains. Rab11 also directly links vesicular trafficking to actin [60, 61] as well as microtubule motors [62]. Furthermore, the Rab proteins Rab10 [63], Rab8 [57, 64] and other Rab family proteins have been implicated in the generation or maintenance of epithelial polarity [65].

Rho family GTPases including Cdc42, Rac1 and RhoA are regulators of the actin cytoskeleton and key players in polarity [43, 66] (Fig. 1a). In addition to its essential role in the PAR complex, Cdc42 is also involved in the regulation of actin dynamics at cell–cell junctions, together with other members of the Rho family of GTPases [43]. Tight junctions and adherens junctions are closely coupled to the actin cytoskeleton, which promotes not only tissue integrity but also cell–cell communication and epithelial remodelling by coordinated actomyosin contraction within epithelia. While Rac1 and Cdc42 promote branched actin structures during initial junction formation, RhoA activity during maturation leads to the formation of a contractile actomyosin array that provides stability to mature junctions and contractile force during remodelling [43]. Although research has mainly focused on the three Rho family proteins Cdc42, Rac1 and RhoA, it is conceivable that other Rho family proteins, their upstream regulators, downstream effectors and interaction partners also play important roles in the regulation of polarity [66–68]. In addition to its function in junctional stability, actin is mostly localised at the apical domain via linker proteins of the ezrin/radixin/moesin (ERM) family that bind actin and PIP2 [69, 70] to form the cytoskeletal core of microvilli.

Not only the actin cytoskeleton, but also the microtubule and intermediate filament cytoskeletons play important roles in the establishment and sustenance of apical–basal architecture. Microtubules in epithelial cells are nucleated from non-centrosomal nucleation sites below the apical surface and are oriented in parallel bundles with the minus ends oriented towards the apical side [71] (Fig. 1a). Directional movement of microtubule motors along these microtubule tracks leads to polarised trafficking important for both establishing and maintaining apico-basal asymmetry [71]. Furthermore, microtubules are important for nuclear positioning [72], PCP and cilia orientation [73] and as components of the mitotic spindle [74]. Mitotic spindle orientation is essential to establish, maintain or reorganise epithelial architecture by ensuring correct alignment of cell divisions within epithelia. Mechanical cues from the local tissue environment are translated into spatial information for spindle positioning by E-cadherin [75], which recruits the leucine–glycine–asparagine repeat protein (LGN) adaptor protein and SCRIBBLE [75–77] to determine the site of astral microtubule attachment to the cell cortex and thus orientation of the mitotic spindle. Consistently, deletion of E-cadherin in prostate epithelium was demonstrated to cause loss of polarity, misorientation of cell division and hyperplasia [76].

Keratins, the main intermediate filaments of epithelial cells are localised to the apical and apico-lateral surface, where they not only provide mechanical stability, but also directly contribute to epithelial polarity [78]. Keratins have furthermore been demonstrated to be involved in the organisation of microtubule-organising centers and microtubules in epithelial cells and may play a role in directional trafficking [46].

All of the above components that establish and maintain epithelial polarity also contribute to other polarised phenotypes that can transition into one another by rewiring of the underlying polarity signalling and interaction networks.

### EMT and epithelial plasticity

EMT is an essential process during embryonic development (type I EMT) and tissue repair (type II EMT) in which epithelial cells lose their inter-cell adhesion and epithelial polarity and morphology and acquire migratory, invasive and stemness features [13, 79]. Type III or oncogenic EMT contributes to progression and metastasis of tumours derived from epithelial origin by enabling tumour cells to detach from the primary tumour and to disseminate to secondary sites. Loss of epithelial polarity can already occur to some extent early in cancer development and may directly be involved in tumorigeneseis [14, 80]. During tumour progression, epithelial polarity is lost and cells adopt mesenchymal and migratory properties, a step marked by downregulation of E-cadherin and upregulation of N-cadherin [81] and by downregulation of keratins and upregulation of vimentin [13]. EMT can be induced or modulated by environmental factors [82] such as transforming growth factor-beta (TGF-β) signalling or mechanical factors, signalling pathways including Wnt, Notch or Hedgehog and cellular expression patterns of EMT-linked transcription factors such as SNAIL, Twist or Zeb and microRNAs (reviewed by [83]). Interestingly, a recent study has demonstrated that intact apico-basal polarity itself can protect organoid 3D cultures from EMT by Par3/aPKC-dependent degradation of the SNAIL-family protein SNAI1 [84].

An association of EMT with tumour progression has clearly been demonstrated by the effects of expression of EMT-related genes on metastasis [85, 86]. Furthermore, increased expression of EMT-linked transcription factors is correlated with poor prognosis in patients [87–89]. However, evidence is mounting that full epithelial-to-mesenchymal conversion is not a prerequisite for all tumour cells to metastasise but may rather be connected with the high plasticity of metastasising cells [90–93], shifting the view on EMT from a stable phenotypic switch to a dynamic range of transitional states [94]. Intermediate states of EMT have been described in collectively invading tumour cells that acquire migratory capacity [95, 96] but undergo incomplete EMT, retain cell–cell contacts and sometimes display very small changes in EMT markers [97].

Consequently, dysfunction or mislocalisation of polarity regulators can also promote metastasis in the absence of EMT by affecting local invasion. For example, combined knockdown of two of the polarity regulators Scribble, Dlg1 or AF-6 or knockdown of one of these polarity regulators in combination with ErbB2 activity [98] as well as knockdown of Par3 in combination with ErbB2 activity [99] leads to loss of epithelial polarity and induces invasion of breast cancer cells without inducing EMT. Downregulation of Par3 also induced invasion and metastasis in oncogenic Notch intracellular domain or H-Ras Q61L mouse breast cancer models [100]. In contrast, downregulation of Par3 reduced invasion and metastasis in a prostate cancer model [101] and led to defective collective migration in A431 squamous carcinoma cells [102]. The example of Par3 perfectly reflects the complexity, versatility and finely balanced regulation of cellular polarity modules and demonstrates how the phenotypic output depends on cancer type, cell type, model system and cellular context. Intermediate states of EMT have also been observed in CTCs, where tumour cells express both, mesenchymal and epithelial markers [103, 104]. Due to their high plasticity, flexibility and adaptability, some metastasising tumour cells might thus be able to bypass anti-metastatic therapies targeting EMT.

### Cell migration modes

During invasion, tumour cells can adopt different modes of migration, ranging from collective migration of cell strands or sheets to loosely connected cell streaming to mesenchymal or amoeboid single-cell migration [8, 16]. The various migration modes have been suggested to represent different levels of epithelial dedifferentiation with collective migration being the least dedifferentiated, mesenchymal migration intermediate and amoeboid migration the most dedifferentiated state [10]. We suggest that single-cell polarity in liquid phase [20] represents an even more dedifferentiated state characterised by uncoupling of the subcellular polarity machineries as discussed below. Like epithelial dedifferentiation, migration modes are inter-convertible. Tumour cells have the ability to switch between different migration modes in response to cell-intrinsic or environmental factors or upon inhibition of one type of migration [105–109], contributing to plasticity and adaptability of metastasising cells. Thus, only a common mechanism that is essential for all possible modes of tumour cell migration could potentially provide molecular targets for the prevention of migratory phases of tumour metastasis [8].

### Front–rear polarity

Irrespective of the mode of movement, migrating cells need to adopt front–rear polarity, which is established and maintained by both changes in cellular expression patterns as well as repositioning and rewiring of the same polarity modules that organise apical–basal polarity (Fig. 1b). The specific functions of these polarity modules during front–rear polarity are described in this section.

In addition to its role in epithelial polarity, the Par complex also plays essential roles in establishing front–rear polarity in migrating cells [110] (Fig. 1b). Active Cdc42 at the cell front recruits Par6 and aPKC, which leads to recruitment of adenomatous polyposis coli (APC) to microtubule ends at the leading edge to stabilise cell polarisation [35, 111, 112]. In addition, Par6 promotes local downregulation of RhoA at the cell front [113, 114]. Par3 can activate Rac1 via the exchange factor Tiam1, which leads to further stabilisation of front–rear polarity [115].

PATJ, a component of the Crumbs complex is localised at the cell front (Fig. 1b) in wound‐healing assays and is required for the correct localisation of Par3 and aPKC and for centrosome orientation [116]. Crumbs proteins can also interact with actin-binding proteins and may cooperate with Rho family GTPases to regulate the actin cytoskeleton [117].

The Scribble complex localises to the front of migrating cells (Fig. 1b), where Scribble increases Rac1 and Cdc42 activity to promote actin polymerisation and formation of cell protrusions [118]. Dlg1 is recruited to the cell front in a Scribble- and Par complex-dependent manner, where it interacts with APC to stabilize microtubule ends [35], while Lgl regulates myosinII and focal adhesion morphology [119].

Components of the PCP complex are involved in various processes during migration and polarity. Non-canonical Wnt signalling is a potent activator of different modes of migration [120]. Most prominently, Wnt5a activates motility and invasion of tumour cells by different mechanisms [120]. Wnt5a promotes persistence in migrating melanoma cells by stable rear polarisation through formation of a structure termed W-RAMP, containing F-actin, myosin IIB, melanoma cell adhesion molecule (MCAM) and the non-canonical Wnt receptor frizzled 3 [121]. Furthermore, Vangl1 acts in a complex with Scribble at the cell front [122] (Fig. 1b) and Vangl 2 has been implicated in the downregulation of metalloproteinases required for protease-dependent invasion [123].

Local activities of PI3Kinase at the cell front and of PTEN at the cell rear establish an asymmetric phospholipid distribution in migrating cells with higher PIP3 concentrations at the front and higher PIP2 concentrations at the rear [124] (Fig. 1b). PIP3 amplifies “frontness” in a positive feedback enhancing Rac1 activity [125, 126] and promotes RhoA-dependent “backness” by activation of Cdc42 [127]. At the rear, PIP2 binds and activates ERM proteins that organise the actin cytoskeleton and activate RhoA signalling necessary for rear retraction [128].

The main role of Rab family members in cell migration is to direct endocytosis, recycling and secretion of membrane and cargo such as integrins or metalloproteinases. Rab5 is a key regulator of early endosome formation and recycling. It controls localisation and activity of integrins at the surface and is directly involved in the formation of protrusions [129]. Rab 25 has been shown to promote invasion by retaining an active pool of *α*_5_*β*_1_-integrin at the tip of invasive pseudopods [130]. Many other members of the Rab family have also been demonstrated to contribute to migratory or invasive behaviour of tumour cells mostly by directing endocytosis, recycling and secretion [131]. To which extent Rab-regulated directed trafficking directly contributes to front–rear polarity and the crosstalk between Rab GTPases and other polarity networks are not fully understood and likely depends on the mode of migration.

Rho family GTPases regulate various aspects of front–rear polarity [132, 133]. At the cell front, actin branching and elongation is required to form cell protrusions and to regulate assembly and disassembly of focal adhesions [134], while at the cell rear, actomyosin-driven contractility creates the necessary force to drive the cell body forward [15, 135]. Both of these processes underlie strict regulation by localised activities of Rho family GTPases [133], with high levels of Rac1 and Cdc42 activity leading to actin polymerisation in the front and high levels of RhoA activity leading to rear retraction (Fig. 1b). Mutual inhibition of Rac-dependent “frontness” and RhoA-dependent “backness” and positive feedback loops [125] can lead to self-organisation of cell polarity in directionally migrating cells [136, 137]. While this model accounts for many aspects of front–back polarity, it simplifies the complex interactions within the RhoGTPase network. RhoA activity for example is not strictly localised to the cell rear, but also locally activated at the leading edge [138, 139]. Furthermore, net activation levels of Rac1 and RhoA also regulate cell plasticity by determining the mode of migration and can induce a morphological switch [107, 140]. Cdc42 is a central regulator of cell polarity and directionality with various functions in migrating cells. In addition to regulating the Par complex and actin polymerisation to establish front–rear polarity, Cdc42 also participates in direction sensing by orienting the microtubule cytoskeleton and aligning the centrosome and nucleus along the front–rear axis [141–143]. Furthermore, local Cdc42 activation antagonises RhoA activity and directs chemotactic steering and polarisation [144].

In migrating cells, the nuclear centrosomal axis is always aligned with the front–rear axis with the centrosome either placed at the front or at the back of the nucleus, depending on cell type, migration mode and matrix properties [145]. The centrosome serves as the microtubule-organising centre (MTOC), which leads to an orientation of microtubule plus ends towards the cell periphery and to an arrangement of microtubules in a parallel array along the front–rear axis facilitating directional vesicle trafficking by microtubule motor proteins for the transport of membrane and cargo to the cell front [146] (Fig. 1b). Specific sets of plus end-binding proteins coordinate local functions of microtubules and crosstalk with the actin cytoskeleton and Rho family GTPases. At the cell front, microtubules contribute to regulation of protrusion and focal adhesion formation, while at the rear they assist focal adhesion disassembly [146].

Also intermediate filaments directly contribute to polarisation in migrating cells [134]. Vimentin has been demonstrated to organise and stabilise the polarised orientation of the microtubule cytoskeleton during directed cell migration [147] and to enhance cell motility and adhesion during EMT [148, 149].

In summary, front–rear polarity is established by physical and functional rearrangement of the same polarity modules and molecular players that constitute epithelial polarity. In addition to front–rear and epithelial polarity, specialised cells can adopt other polarised phenotypes during essential biological processes such as asymmetric cell division, neuronal differentiation or establishment of the immunological synapse, which are discussed in detail elsewhere [9, 150]. The underlying extracellular and intracellular and signalling processes that control different types of polarity as well as crosstalk within the cellular polarity machinery have recently been thoroughly and comprehensively reviewed [9, 10]. In the following sections, we will, therefore, mainly focus on the mode of polarisation of tumour cells during circulation and early seeding, on the role that polarisation of CTCs may play in the metastatic process and how it could be targeted therapeutically.

### Single-cell polarity in liquid phase

We have recently demonstrated that tumour cells maintain a basic cortical polarisation in liquid phase in vitro and in vivo, termed single-cell polarity in liquid phase [20]. In general, the actin cortex and the plasma membrane are organised by ERM-family and related proteins such as Merlin, the product of the tumour suppressor neurofibromatosis type II (NF2) [69]. Single-cell polarity (Fig. 1c) is characterised by an asymmetric distribution of cortical actin, plasma membrane, ERM proteins, phosphatidylinositol 4,5-bisphosphate (PIP_2_) and transmembrane proteins such as integrins or cell adhesion molecules. The plasma membrane is strongly folded at the pole, increasing the cellular surface at this site (Fig. 1c). In composition and localisation of molecules, single-cell polarity is similar to the polarisation of amoeboid migrating cells [135, 151, 152]. However, unlike in amoeboid migrating cells, nucleus and centrosomes are not aligned with the single-cell pole [20], indicating that the polarisation of the plasma membrane and the actin cytoskeleton is not coupled to Cdc42- and microtubule-based centrosome positioning and intracellular polarity. We, therefore, suggest that single-cell polarity constitutes a generic type of polarity of the actin cortex, linker proteins and plasma membrane that tumour cells maintain over hours in suspension after uncoupling from the intracellular polarity machinery.

While epithelial polarity and front–rear polarity are regulated and maintained by common sets of regulatory factors and polarity complexes [9, 10], it is still unclear whether components of polarity complexes such as PAR, CRUMBS, SCRIBBLE or PCP and other polarity regulators play a role in the regulation of single-cell polarity in liquid phase. However, we have demonstrated that the apical polarity marker podocalyxin does not colocalise with the cortical ezrin-rich pole [20]. Although a fraction of cells in suspension displayed polarised podocalyxin, the site of podocalyxin accumulation did not colocalise with the ezrin-rich pole. This suggests that cortical polarity can co-exist in a cell with apical polarity as two distinct, uncoupled polarity circuits. The contribution of other polarity regulators to single-cell polarity in liquid phase remains to be investigated. However, given the essential roles of Rho family GTPases as actin cytoskeleton organisers and polarity regulators [133, 141, 153], it is likely that they are also involved in establishing or stabilising single-cell polarity.

### (Un-) coupling of cortical and intracellular polarisation

We propose that single-cell polarity in liquid phase constitutes a type of basic cortical polarity that can be stably maintained until the cell receives a directional cue that triggers coupling to the intracellular polarity machinery and transition to a fully polarised phenotype. It has been demonstrated in different organisms and cell types that cortical polarity can be maintained in the absence of microtubule function but that microtubules are necessary for the positioning of cortical polarity, coupling to the intracellular polarisation machinery and persistence of polarised phenotypes [154]. RPE1 or NIH 3T3 cells treated with the microtubule-depolymerising agent nocodazole maintain their polarisation and ability to migrate directionally, but with decreased persistence of migration direction [155]. Also, fast, directional movement of fish keratocytes or cytoplasmic fragments is microtubule-independent [156]. In neutrophils, disruption of microtubules can even enhance polarisation and migration [157], although nocodazole treatment reduces the chemotactic efficiency of neutrophils [158]. Altogether, these studies demonstrate that actin-based cortical polarity can be uncoupled from the microtubule-based, directional polarity machinery.

Initial cortical polarisation can be triggered by external or cell-intrinsic cues such as sperm entry, cell–cell interactions, cell–matrix interactions or cell division scars [159, 160]. In various organisms, cell types, and biological processes, microtubules can generate cortical polarity [154], but they may not be the initiating factor in de novo polarisation processes [161]. In the well-studied polarisation events during early embryo development, cortical polarisation is closely coupled and interconnected with centrosome positioning, which seems to be the initiating signal [161, 162], and with the Rho family GTPase network and PAR proteins, which are segregated into distinct domains by asymmetric actomyosin contractility and activation of Rho proteins [162, 163]. A recent study showed that actomyosin contractility is not necessarily the initial polarisation event, as in spontaneously differentiating epithelial MDCK cells, the Cdc42 effector MRCK acts upstream of actomyosin contractility to initiate apical polarisation [164]. In all of these polarisation processes, cortical actomyosin polarity is closely linked to the other polarity machineries in the cell in an interconnected network of reciprocal regulation.

In contrast, single tumour cells can undergo processes where cortical polarisation is initially established or maintained until an additional trigger connects it to other polarity complexes in the cell [20, 21, 165]. Little is known about the cues, underlying mechanisms and molecular linkers involved in establishing or maintaining initial cortical polarity and coupling cortical polarity to the intracellular polarity machinery in human tumour cells.

Evidence for a potential coupling mechanism comes from a study investigating cortical polarisation and its connection to centrosome positioning in single, dividing Caco-2 cells embedded in a three-dimensional matrix [165]. McClatchey and colleagues observed that prior to the first cell division, ezrin and actin concentrate in a cap-like structure at one pole of single cells, by a cell-cycle-dependent, microtubule-independent mechanism. They found that α-catenin-dependent cortical localisation of Merlin is necessary to restrict cortical ezrin to a single pole and to position the centrosome beneath the ezrin cap. The microtubule-binding protein adenomatous polyposis coli-like molecule 2 (APC2) was identified as a linker protein required for the association between ezrin cap and centrosome. In SkMel2 melanoma cells, knockdown of NF2 also reduced single-cell polarity in liquid phase, attachment, adhesion, transmigration and metastastic seeding [20], although the effects were cell line-specific. A role for APC2 in coupling cortical polarity to centrosome positioning has not yet been investigated in cells transitioning from liquid phase to an adhered state. In another study, Sanchez-Mateos and colleagues have demonstrated that previously unpolarised melanoma cells undergoing early adhesion form a cortical cap containing the ERM protein moesin and actin [21]. They identified moesin (but not ezrin) as a key regulator of RhoA activation and subsequent myosin II contractility in response to attachment, demonstrating a link between cortical polarity and the Rho family GTPase network.

Other examples for possible mechanisms that might play a role in coupling cortical polarity to intracellular polarity regulators include cytoskeletal integrators that connect different types of cytoskeletons such as CLASPs and Spectraplakins [166, 167], myosin 10 [168–170], direct interactions between polarity complex components and ERM proteins such as between Crumbs and Moesin in Drosophila [171, 172], or even direct interaction of microtubules with plasma membrane domains as observed in fission yeast [173]. In an in vivo situation, most likely more than one of these mechanisms will be involved and work in a concerted fashion to achieve a fully polarised phenotype.

It is still unresolved whether there is a general underlying principle to connect different cellular polarity machineries. Although the same network of molecular players is involved, the hierarchy and topology of the polarity network seems to be flexible. Cells certainly employ highly individual mechanisms depending on the specific polarised phenotype, environmental factors or the cell type. How such mechanisms can be targeted therapeutically remains to be investigated. Future studies on the molecular players and the coupling of different types of cellular polarity under various physiological conditions will hopefully shed new light on these questions.

## Polarisation of CTCs

### Characteristics and mechanisms of single-cell polarisation

We have recently demonstrated that CTCs isolated from the blood of cancer patients display the same cortical polarity features as tumour cells in supension in vitro, including polarised accumulation of ezrin, actin, CD44, MCAM or beta1-integrin [20]. It is unclear whether single-cell polarity in CTCs constitutes residual polarity maintained upon dissociation from primary tumour, intravasation or dissociation of CTC clusters or whether single CTCs can acquire de novo cortical polarity from an unpolarised state.

In vitro, tumour cells in suspension can maintain a stable single-cell pole as remnant of a previous polarised state for hours but rarely newly acquire a single-cell pole by symmetry breaking [20], which is consistent with the observation that spontaneous symmetry breaking in tumour cells requires adhesion signalling [174]. However, in the in vivo situation, CTCs are exposed to external cues that can trigger polarisation such as collisions and interactions with immune cells and platelets [175–177], shear stress [178], or confinement in small vessels [179, 180]. It is, therefore, reasonable to assume that CTCs can newly establish a single-cell pole during circulation.

### Polarity in CTC clusters

CTCs can circulate in the blood not only as single cells but also as multicellular CTC clusters [181–183] detached from primary tumours [182, 184]. CTC clusters often contain non-tumour cells and have been associated with increased metastatic potential in model systems and worse prognosis in cancer patients [182, 185–187]. Currently, only limited data are available on the polarisation of CTCs within clusters.

CTC clusters have undergone partial EMT, losing epithelial architecture and most likely polarity, while maintaining strong cell–cell connections [104, 182, 184, 187]. A study investigating expression and localisation of EMT markers in single CTCs and CTC clusters isolated from the blood of lung cancer patients revealed loss of plasma membrane E-cadherin as well as expression of vimentin in most CTC clusters [187]. However, expression of cytokeratin as well as N-cadherin was also observed. Expression patterns were very heterogeneous between patients and clusters and even within clusters. Maheswaran and colleagues [104] found that CTC clusters isolated from the blood of breast cancer patients strongly expressed mesenchymal markers and only weakly expressed epithelial markers [104]. At the same time, CTCs in clusters express the epithelial marker keratin 14 [184] and the cell–cell junction protein plakoglobin [182], both of which are directly associated with cluster formation. More detailed investigation of the interconnections and functions of polarity components within CTCs in clusters will be necessary to define the types and possible variations of polarity occurring in CTC clusters.

The relationship between the prevalence of CTC clusters and single-cell polarity in single CTCs in cancer patients or model systems has not been investigated. It could be speculated that CTC clusters constitute a source of strongly polarised single CTCs. Due to the strong cell–cell connections providing polarity cues within CTC clusters, single CTCs either shedded from clusters in circulation or mechanically dissociated during CTC isolation might display higher single-cell polarity.

### Implications of single-cell polarity in CTCs for metastasis

**Fig. 2:**
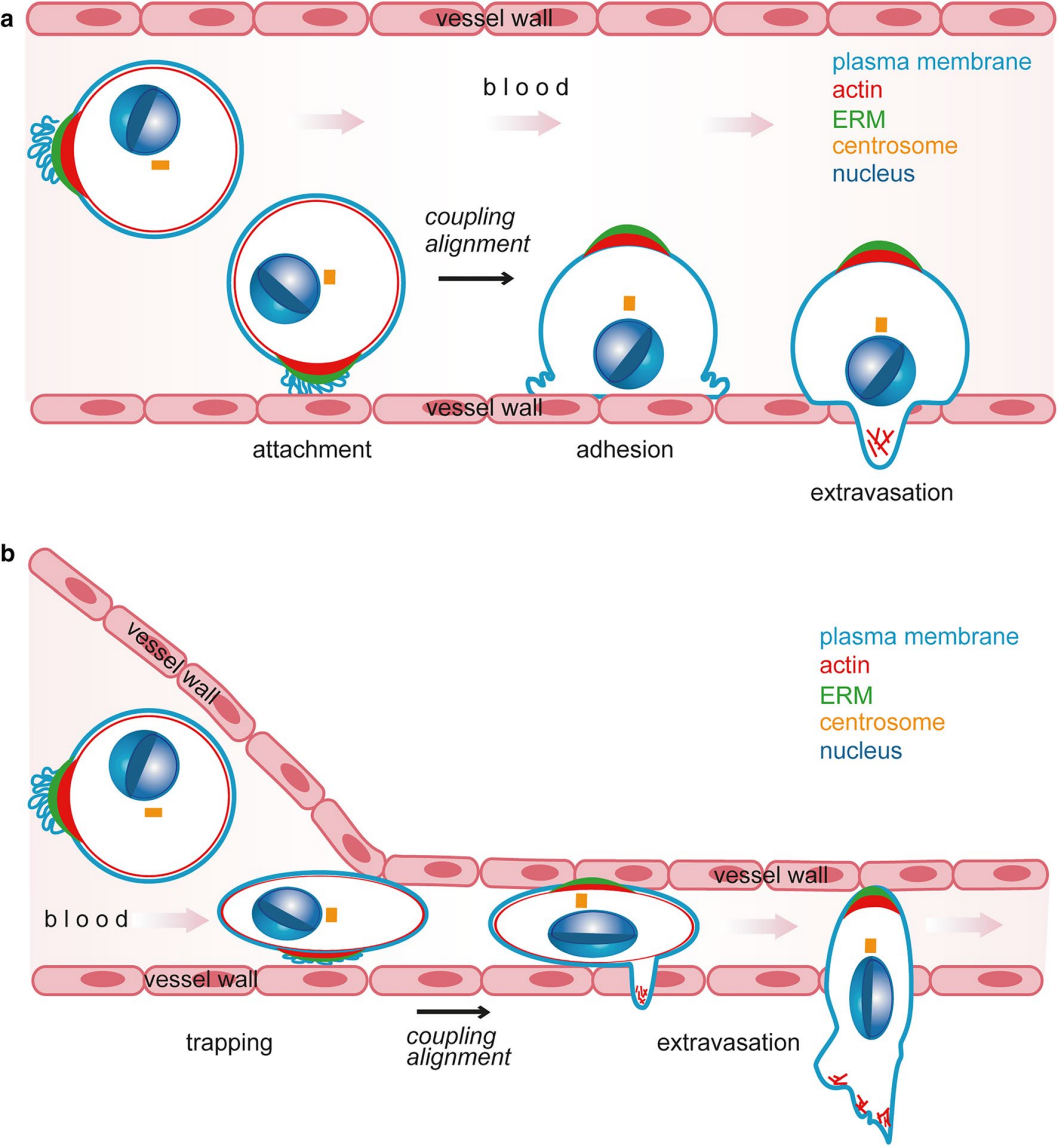
Exit of CTCs from the circulation. **a** Attachemnt, adhesion and extravasation of CTCs in larger vessels. CTCs can attach to the endothelial wall of blood vessels. During active adhesion to the vessel wall, cortical single-cell polarity is coupled to intracellular polarity modules, leading to nucleo-centrosomal alignment necessary for migration and extravasation. **b** Mechanical trapping and extravasation of CTCs in smaller vessels. CTCs get passively arrested in blood vessels of small diameter. Similar to **a**, extravasation and migration require coupling of cortical and intracellular polarity modules and nucleo-centrosomal alignment

Cortical polarisation provides CTCs with an advantage during metastatic seeding. We have demonstrated that single-cell polarity correlates with the metastatic capacity in mouse models for CTCs, and with the metastatic potential of human tumour cell lines [20]. Furthermore, inhibition of single-cell polarity either by manipulation of expression levels of single-cell polarity regulators or generic depolarisation reduced metastatic seeding in mice. Single-cell polarity thus constitutes a metastatic quality of CTCs. In vitro, single-cell polarity affects two cellular properties that could contribute to metastatic seeding of CTCs, early attachment and adhesion [20] (Fig. 2). Early attachment may be reinforced by local accumulation of plasma membrane and highly glycosylated proteins at the pole that generate a “sticky” end with a high tendency for unspecific interactions with a substrate. Initial contact of the pole with endothelium will establish close proximity of membrane receptors and ligands to favour the establishment of receptor-mediated interactions between tumour cell and endothelium. Adhesion could be advanced by cortical polarity serving as “pre-polarisation” of the plasma membrane and actin cytoskeleton. During attachment, polarised cells do not require symmetry breaking but only coupling of their already established cortical polarity to the intracellular polarity machinery. An in silico model supporting this theory predicted a threefold attachment rate and a 1.3-fold adhesion rate for polarised cells as compared to unpolarised cells [20]. However, these mechanisms remain to be confirmed by intravital imaging.

During metastasis, both enhanced attachment and adhesion can favour extravasation of CTCs. In circulation, tumour cells are no longer protected by the immune-suppressive environment of the primary tumour against attacks by immune cells [188] and become exposed to shear stress by blood flow [189]. Therefore, the probability of a CTC to successfully survive, seed to a distant site and initiate metastasis is highly dependent on attachment, adhesion and extravasation to escape the hostile environment of the blood circulation [190].

**Fig. 3:**
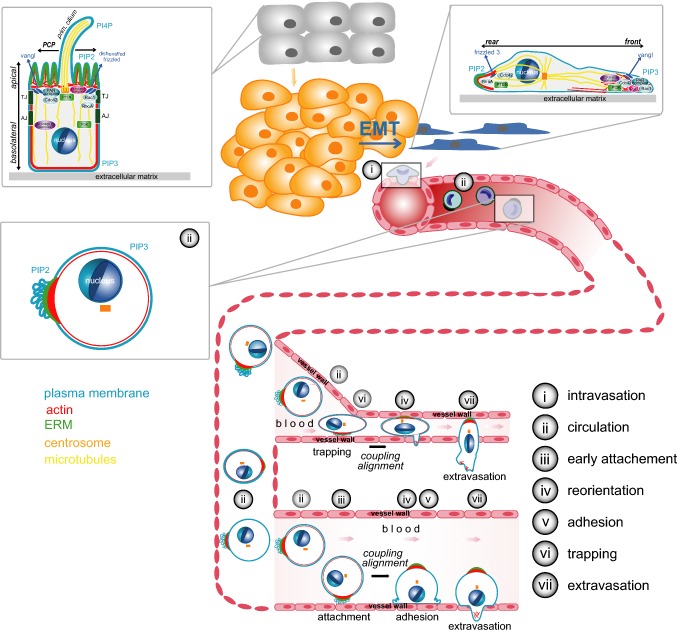
Types of polarity during tumour progression. Overview of the process of carcinogenesis and metastasis (adapted from [20]). Insets show the steps at which specific types of polarity are involved. Healthy cells (grey) can accumulate mutations leading to uncontrolled growth, evasion of cell death and development of a tumour (yellow). Tumour cells can undergo EMT (yellow to blue), leading to further dedifferentiation and shift to a migratory phenotype (blue) favouring invasion, migration and intravasation into the lymph or blood circulation. Circulating tumour cells can exit the circulation by mechanisms shown in detail in Fig. 2. For further details see Figs. 1, 2 and main text

In vivo, two mechanisms can promote the arrest of CTCs: active attachment to the wall of vessels with a larger diameter [191, 192] or passive confinement of CTCs in small vessels [193, 194] (Figs. 2, 3). An increased attachment rate due to single-cell polarity will thus enhance attachment of CTCs to larger vessels (Fig. 2). In the case of arrest by confinement, blood pressure counteracts adhesion of arrested cells and leads to recirculation of CTCs [195]. Cortical “pre-polarisation” of a cell will advance adhesion and thereby reduce the probability of recirculation (Fig. 2). Whether single-cell polarity also contributes to the metastatic potential of CTCs by additional mechanisms or whether single-cell polarity reflects other metastatic features of CTCs remains to be investigated.

## Clinical potential of single-cell polarity

In order to exploit the discovery of single-cell polarity as a metastatic feature of CTCs for diagnostic or therapeutic purposes, the molecular details and regulatory processes underlying the establishment and maintenance of single-cell polarity as well as the connection of cortical polarity to other cellular polarity machineries need to be identified. We have previously described that expression of the known potential prognostic and therapeutic targets melanoma cell adhesion molecule (MCAM) [196], L1 adhesion molecule (L1-CAM) [197] or intercellular adhesion molecule 1 (ICAM-1) [198] also affects single-cell polarity. The objective should now be to identify novel regulators of single-cell polarity that can either be used as prognostic markers or constitute therapeutic molecular targets to interfere with metastatic seeding of CTCs. Exploiting single-cell polarity for diagnostic or therapeutic use may have several advantages over other steps of the metastatic cascade. Rapid technological advances in the analysis of liquid biopsies [199–201] make markers expressed by CTCs especially accessible for diagnostic use with minimally invasive procedures for patients. Moreover, targeting tumour cells in liquid phase in the blood would improve the accessibility of therapeutic agents. Single-cell polarity regulators furthermore have the potential to provide broad targets, independent of tumour entities or individual, tumour type- or subclone-specific mutations. Although we have to acknowledge that inhibition of single-cell polarity will not eradicate metastasis, inhibition of the exit of CTCs from the blood circulation may reduce the probability of formation of distant-organ metastases for the benefit of cancer patients. Further investigations into the underlying mechanisms and regulators controlling single-cell polarity may thus provide promising targets for future therapies of metastatic cancer.

## Perspectives

The discovery of single-cell polarity in liquid phase has added an additional element to our understanding of the varying roles that components of cellular polarisation systems can play during tumour progression and metastasis, but many open questions remain. The next steps will have to address the detailed underlying principles on a molecular and cell biological level and the implications for the area of cancer cell polarity. To determine whether single-cell polarity could indeed constitute a direct target for future anti-metastatic therapy, the clinical connection between polarisation of CTCs from cancer patients and metastasis needs to be investigated. This major open question will now have to be approached in large clinical studies. Another task will be to decipher the interconnections of single-cell polarity with other types of cell polarity. It is still unclear whether pre-polarisation by single-cell polarity favours the adoption of any fully polarised phenotype or whether it primes cells towards a more epithelial or more mesenchymal phenotype with all the implications this may have for their metastatic capacity. Finally, the question remains as to what distinguishes a polarised cell from an unpolarised cell on the molecular level. Insights into these differences will hopefully lead to the discovery of regulators of single-cell polarity as potential prognostic markers or molecular targets for anti-metastatic therapies. Addressing these open points will lead to a deeper understanding of the complex functional interactions and consequences of deregulation of polarity components on cancer metastasis and, eventually, how this knowledge can be exploited for the development of anti-metastatic therapies.
